# Classification of Genes: Standardized Clinical Validity Assessment of Gene–Disease Associations Aids Diagnostic Exome Analysis and Reclassifications

**DOI:** 10.1002/humu.23183

**Published:** 2017-02-13

**Authors:** Erica D. Smith, Kelly Radtke, Mari Rossi, Deepali N. Shinde, Sourat Darabi, Dima El‐Khechen, Zöe Powis, Katherine Helbig, Kendra Waller, Dorothy K. Grange, Sha Tang, Kelly D. Farwell Hagman

**Affiliations:** ^1^ Ambry Genetics Aliso Viejo CA 92656; ^2^ Department of Pediatrics Washington University School of Medicine, St. Louis Children's Hospital St. Louis MO 63110

**Keywords:** diagnostic exome sequencing, clinical validity, characterized genetic etiology, gene–disease association, SCYL1, SNAP25, novel candidate genetic etiology, reclassifications

## Abstract

Ascertaining a diagnosis through exome sequencing can provide potential benefits to patients, insurance companies, and the healthcare system. Yet, as diagnostic sequencing is increasingly employed, vast amounts of human genetic data are produced that need careful curation. We discuss methods for accurately assessing the clinical validity of gene–disease relationships to interpret new research findings in a clinical context and increase the diagnostic rate. The specifics of a gene–disease scoring system adapted for use in a clinical laboratory are described. In turn, clinical validity scoring of gene–disease relationships can inform exome reporting for the identification of new or the upgrade of previous, clinically relevant gene findings. Our retrospective analysis of all reclassification reports from the first 4 years of diagnostic exome sequencing showed that 78% were due to new gene–disease discoveries published in the literature. Among all exome positive/likely positive findings in characterized genes, 32% were in genetic etiologies that were discovered after 2010. Our data underscore the importance and benefits of active and up‐to‐date curation of a gene–disease database combined with critical clinical validity scoring and proactive reanalysis in the clinical genomics era.

## Introduction

Whole‐exome sequencing (WES) has rapidly moved from the research domain into the clinical setting. In the last few years, WES has played an increasing role in healthcare and has become an important line of inquiry in diagnostic medicine. For patients, families, clinicians, and payers, identification of a molecular diagnosis can end the heavy burden imposed by the “diagnostic odyssey” of expensive, invasive, time‐consuming testing and can potentially lead to changes in patient care [Soden et al., 2014; Srivastava et al., 2014]. For researchers and diagnostic laboratories, the widespread use of diagnostic exome testing has also provided vast amounts of data that can be used to refine the method further and fuel genetic discovery. The last decade saw the continual improvement and standardization of variant pathogenicity criteria for clinical reporting [Richards et al., 2015; Amendola et al., 2016]. The advent of diagnostic exome sequencing (DES) begets the need for a similar standardized system for gene characterization (i.e., “clinical validity assessment”). At the end of the human genome project in 2003, the number of Mendelian genes with a known phenotype or a reported disease‐causing variant was 1,474, and by 2013, the number had doubled to 2,972 (according to NIH estimates [National Human Genome Research I, 2013]. As of October 2016, OMIM catalogs 3,638 genes with a reported phenotype‐causing variant [Medicine M‐NIoG, 1966–2016]. There are still many gene–disease relationships left to discover, however; Cooper et al. (2010) estimated that there are about 5,000–10,000 undiscovered disease genes, and Chong et al. (2015) suggested that there are about 9,000 disease genes remaining to be characterized. These estimates of the number of gene–disease relationships still undiscovered are further complicated by genetic heterogeneity and pleiotropy. Despite the large number of uncharacterized genes, current WES diagnostic rates average about 30% among characterized genes [Yang et al., 2013; Lee et al., 2014; Yang, et al., 2014; Farwell et al., 2015; Retterer et al., 2015; Lazaridis et al., 2016], and this rate is expected to increase over time as knowledge of the human genome increases [Biesecker and Green, 2014]. This prediction is supported by our experience at that roughly 23% of positive findings from our first 500 DES cases were in genes in which the associated disease was discovered within the previous 2 years [Farwell et al., 2015]. DES has emerged as a valuable clinical tool currently, and will have increasing value as new gene–disease relationships are elucidated.

A major challenge for diagnostic laboratories is interpreting the clinical validity of a gene–disease association, defined in Biesecker and Green (2014) as “the determination that a particular disease is truly caused by variants in a particular gene and that the specific variant that has been detected is indeed pathogenic.” Prior to the public release of large databases of control populations such as ExAC [Lek et al., 2016], many genes with ethnicity‐specific benign variants detected in patients were reported as candidate disease genes. As more control genomes are sequenced, however, they reveal genes with high tolerance for variation. For instance, in light of the National Heart, Lung, and Blood (NHLBI) Exome Sequencing Project, Piton et al. (2013) systematically reassessed 106 genes previously implicated in X‐linked intellectual disability (XLID). The authors found that at least 15 genes needed confirmatory studies to be confidently associated with XLID, and in particular they highly questioned the involvement of an additional 10 genes in XLID. More recently, gene‐specific constraint scores have been published using the ExAC dataset of typically developing individuals expands number of genes with questionable pathogenicity [Lek et al., 2016].

Clinical exome or genome sequencing requires interpretations at multiple levels to be clinically valuable. Before the assessment of the pathogenicity of an alteration, it must be determined whether a gene has sufficient evidence to support its involvement in Mendelian disease, that is, whether a gene is “characterized.” Therefore, moving forward, clinical genomics laboratories need to establish strict, consistent criteria to critically examine the strength of gene–disease associations and adopt standardized reporting criteria. A diagnosis based on inadequate evidence has the potential to cause harm to the patient in the form of inappropriate interventions and missed opportunities to find the correct diagnosis. Therefore, just as standardized quality control measures are essential in any other diagnostic test, they are also needed for genetic diagnostics.

Guidelines published by the American College of Medical Genetics and Genomics (ACMG) and the Association for Molecular Pathology have been invaluable for analyzing pathogenicity of specific variants in genes with established causal role for a Mendelian phenotype [Richards et al., 2015]. However, guidelines for assessing the association of a specific gene with a specific disease are still nascent. The Clinical Genome Resource, ClinGen, is coordinating expert analysis of gene–disease associations using a comprehensive and publicly available spreadsheet (https://clinicalgenome.org/working-groups/gene-curation/projects-initiatives/gene-disease-clinical-validity-scoring-matrix/) [ClinGen, 2016]. In diagnostic exome laboratories, however, there remains an urgent need for a simplified scoring system that can be used to systematically evaluate the evidence for both new gene discoveries and previously reported gene–disease relationships. Combining personal communications with the curators of ClinGen, guidelines from MacArthur et al. (2014), and our experience analyzing data from DES of more than 3,000 independent patients plus families since 2011, herein we propose standardized criteria to assess clinical validity of published and potential gene–disease associations. In particular, our scoring system focuses on discriminating whether enough evidence has been accumulated to consider a gene “characterized” for a Mendelian disease and to report to patients as established gene–disease relationships. These criteria are helpful for curating a gene–disease database for both exome sequencing and development of diagnostic gene panels. Furthermore, we also review statistics of patient reclassification reports based on updated clinical validity assessments from the first 4 years of DES at Ambry Genetics.

## Materials and Methods

### Exome Sequencing

Exome sequencing and bioinformatics processing were performed as previously described [Farwell et al., 2015]. Briefly, patient samples were prepared using either the SureSelect Target Enrichment System (Agilent Technologies, Santa Clara, CA), SeqCap EZ VCRome 2.0 (Roche NimbleGen, Mason, WI), or the IDT xGen Exome Research Panel V1.0 (Integrated DNA Technologies, Coralville, IA). Sequencing was performed using paired‐end, 100 or 150‐cycle chemistry on the Illumina HiSeq or NextSeq (Illumina, San Diego, CA). Bioinformatics filtering removed common benign variants, intergenic and 3′/5′ UTR variants, intronic variants outside ±2, and nonsplice‐related synonymous variants. Alterations that were previously classified as pathogenic or likely pathogenic and those that have an HGMD [Stenson et al., 2014] accession number were protected from filtering. Family history‐based filtering and inheritance models were applied to the data, and identified candidate alterations were subsequently confirmed using automated fluorescence dideoxy sequencing.

### Ethics Approval and Consent to Participate

All HIPAA‐covered patient identifiers were removed. Solutions Institutional Review Board determined the study to be exempt from the Office for Human Research Protections Regulations for the Protection of Human Subjects (45 CFR 46) under category 4. Retrospective data analysis of anonymized data exempted the study from the requirement to receive consent from patients.

### Scoresheet for Assessing Clinical Validity

#### Patients and publications

**Figure 1:**
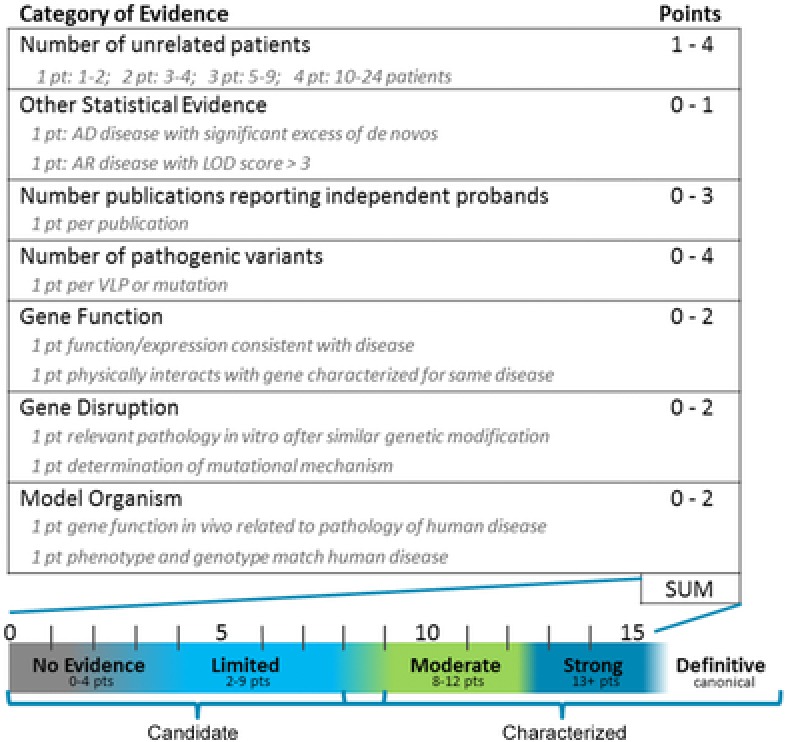
Clinical validity scoresheet for analyzing a gene–disease relationship. Each category of evidence is allowed a maximum specific number of points. Points within each category are summed for a single gene–disease relationship to determine the clinical validity. Patients: one point for one to two patients, two points for three to four patients, three points for five to nine patients, and four points for 10–24 patients. One additional point may be given under “other” for extensive cosegregation (AR disease) or a significant excess of de novo alterations (AD disease). At least one human patient with a rare alteration is required to use this scoresheet. Publications: one point per publication reporting independent probands, up to three points. Variants: one point per pathogenic/likely pathogenic variant reported in a patient, up to four points. Gene function: one point if the gene function and/or expression is consistent with disease phenotype, and second point if gene product physically interacts with a gene product implicated in similar disease. Gene disruption: one point if in vitro experiments show the same disease pathology after a similar genetic modification, and second point if mutational mechanism of patient‐reported alterations is determined by functional studies. Model organism: one point if gene function in an animal model is similar to the pathology reported in the human disease, and second point if both phenotype and genotype of the animal model match human disease. The sum of points for a gene–disease relationship is compared with the scale to determine the final clinical validity category. Gene–disease relationships that are risk association alleles, have no reported evidence, or a score of limited are all considered uncharacterized candidate genetic etiologies. Gene–disease relationships that are moderate, strong, or definitive are characterized genetic etiologies.

Our clinical validity assessment is based on a point system, with points assigned to the relationship between one gene and a single Mendelian disease. When the clinical validity of the relationship between a gene and a specific disease is assessed, the strongest evidence comes from previously reported patients with pathogenic alterations specifically disrupting that gene (Fig. 1). Points are assigned for the number of previously reported, unrelated patients published in peer‐reviewed studies or submitted to ClinVar, and from our internal patient database who received a report with a candidate in an uncharacterized genetic etiology. One point is given for the first two patients, two points for three to four patients, three points for five to nine patients, and four points for 10–24 patients. More than 25 patients reported without conflicting reports will generally lead to a score of “definitive.” Only patients with intragenic alterations may be counted; the effects of an alteration involving multiple genes (such as a complex rearrangement or large deletion or duplication) are not clearly attributable to either single gene. We cap the number of points available for patients at four for two main reasons. First, this rule limits the weight of additional reported patients that may be identified mainly due to ascertainment bias, that is, researchers may perform targeted sequencing of that gene on the patient population and miss alternative possibly explanatory alterations in other genes. For instance, the association between alterations in *RBFOX1* (MIM# 605104) and autism/developmental delay is limited at best, despite the 10+ intragenic deletions reported in patients with autism [Kamien et al., 2014]. Second, simply identifying additional patients does not necessarily contribute to the knowledge of mutational or disease mechanism, which is required to extrapolate the pathogenicity of detected alterations in future patients. Therefore, we prioritize points for other evidence over additional patients.

The scoresheet also allows one point for strong statistical genetic evidence supporting a gene–disease relationship, and the criteria depend on inheritance. Unrelated patients from large cohort papers can be counted as individual proband evidence if the authors present specific phenotypic information, the reported variant is rare in control populations, and statistical evidence from cohort studies is consistent with pathogenicity. Large international cohort papers can also provide valuable statistical evidence for gene–disease association based on aggregate data in addition to individual patient reports. Therefore, we allow an extra point for the identification of a genome‐wide significant excess of de novo alterations in a large cohort exome or genome study, as previously described [Samocha et al., 2014] and recently leveraged in the Deciphering Developmental Disorders (DDD) cohort [McRae et al., 2017]. For an autosomal‐recessive disorder, all patients that are related as second cousins or closer, or who come from an extensive consanguineous pedigree are counted as one independent patient. An extra point can be assigned to large families with convincing cosegregation data for an extremely rare alteration, for instance, with a quantification of genetic linkage (LOD score) of at least 3.0.

One point is given for each publication reporting independent probands with similar phenotypic findings. Publications are carefully assessed to look for patient population overlap. We assign points for the first three independent publications, as a way of measuring independent replication and acceptance for a gene–disease association in peer‐review process. The scoresheet does not assign any points for the time since publication; while newer publications may lack time to refute claims, the increasing yield of diagnosis from Next Generation Sequencing (NGS) and DES has led to many convincing reports published recently.

#### Variant pathogenicity

The scoresheet allows one point for each pathogenic/likely pathogenic variant reported in a patient. Up to four points can be assigned for pathogenic and likely pathogenic variants in patients with the relevant phenotype [Laduca et al., 2014; Pesaran et al., 2016]. This criterion is independent of the number of reported patients or inheritance model, but reflects the fact that variants classified as pathogenic or likely pathogenic are more likely to have a measurable effect on gene function than variants of unknown significance. Patients undergoing exome testing are likely to have many variants of uncertain significance, but have far fewer pathogenic/likely pathogenic variants. Multiple pathogenic variants found in AR disease‐associated genes earn only one point unless they are confirmed to be in *trans*, in which case they can earn a second point. This category allows us to give more weight to alterations more likely to cause the patient's symptoms.

#### Experimental evidence

Up to six total points can be earned for experimental evidence, two points possible in each of three categories: gene function, gene disruption, and model organism. Under gene function, one point may be assigned if biochemical experiments show that the gene function and/or expression are consistent with the disease phenotype. One point may be given if the gene product (mRNA or protein) has been shown to physically interact with or has the same biochemical function as other gene products implicated in a similar disease. Within gene disruption, one point can be given if in vitro experiments show the same disease pathology after a similar genetic modification. A second point can be assigned for experiments that determine molecular mechanism, that is, through phenotype rescue of variant‐expressing cells with the expression of wild‐type sequence (loss of function), or through gene–dosage experiments that establish dominant negative or gain‐of‐function mechanism. Within the category of model organism, one point can be earned if the genetic disruption results in cellular or molecular pathology resembling the human disease. A second point can be earned within model organism if both the phenotype and genotype described in the animal matches human disease, for example, knockout animals have the same phenotype as patients with biallelic loss of function variants.

The points for experimental evidence are informed by the guidelines presented by MacArthur et al. (2014). Our scoring system allows one point for each specified experiment or type of evidence in a yes/no paradigm, which has the benefit of producing consistency between different curators. We have explicitly defined the type of experiment required to assign each integer point, based on how useful that information will be for analyzing variants detected in patients undergoing clinical genetic testing in the future.

### Clinical Validity Categories

The total number of points is used to determine the clinical validity score, as depicted in Figure 1. Gene–disease relationships can be scored as no reported patients (0–4 points), limited (2–9 points), moderate (8–12 points), strong (13+ points), or definitive, similar to the ClinGen gene curation categories. A gene–disease relationship can only be scored as limited or above if there is at least one human patient reported. The category of definitive is generally reserved for Mendelian gene–disease relationships that are established beyond a doubt, are commonly known, or have been reported in at least 25 unrelated patients with rare intragenic variants.

The sum of points is calculated and weighed against any available contradictory evidence, such as evidence of reduced penetrance or the presence of all reported variants in control populations. When scoring a gene–disease relationship, simply omit the specific piece of evidence for which there is contradictory information. For instance, for a published patient with a variant that is too common in control populations to cause disease, exclude that patient, variant, and publication from scoring. The scoresheet is intended for use in highly penetrant Mendelian diseases, and the presence of such contradictory evidence suggests that more information regarding the gene‐disease relationship is required. Extra care must be used when assessing the clinical validity of low‐penetrance genes, and using the present scoresheet may not be appropriate. An additional challenge is encountered when scoring patients with relatively common disorders, such as autism spectrum, or in genes with no clear diagnostic criteria, such as many of the newly characterized neurodevelopmental genes. The category of risk association allele may be a better fit for genes in which variants are merely detected more often in patients than controls, often described with low odds ratios in genome‐wide association studies. In these cases, a thorough literature search is required to distinguish between a gene that causes a Mendelian disease with incomplete penetrance versus a gene that is primarily a risk factor.

The point ranges overlap slightly to reflect the importance of using clinical judgement in cases that are on the border between two categories. For instance, it may be possible to accumulate enough points to consider a gene–disease association as either limited or moderate without the knowledge of the mutational mechanism. Perhaps three independent journal articles reported two de novo predicted pathogenic alterations in three independent families with the same disease (this occurs in autism frequently). The resulting score of eight could fit into either limited or moderate categories. In such a case, it might be prudent to score the gene–disease association as limited until a disease mechanism can be established.

### Evaluating the Performance of This Clinical Validity Scoring System

There is no static “true” score against which to compare the performance of this clinical validity scoring system, nor is there an existing “gold standard” for quantifying clinical validity, so it is impossible to calculate a positive or negative predictive value. Instead of calculating sensitivity and specificity, the strength of the clinical validity scoring system might be evaluated based on social research measures including content validity, concurrent validity, discriminant validity, and consistency. Content validity refers to the inclusion of relevant criteria, and in this case is supported by alignment with previously published guidelines for assessing gene–disease relationships [MacArthur et al., 2014]. Of course, content validity could be increased by operationalizing additional gene–disease information, but this might be achieved at the expense of ease‐of‐use of the scoring system. Concurrent validity refers to the scoring system's ability to distinguish between two groups that it theoretically should be able to distinguish. In the case of gene–disease relationships, a useful scoring system has the ability to discriminate the strength of gene–disease relationships across a broad range of evidence. Discrimination validity refers to the ability to accurately describe variation in strength between neighboring levels. The difference between gene–disease relationships that score limited versus moderate is an important example of discrimination validity in DES, and will be discussed further below when considering characterized genes.

As a measure of scoresheet reliability, three trained independent curators scored the clinical validity for a set of gene–disease relationships from the ClinGen Website as of October 2016 (https://clinicalgenome.org/working-groups/gene-curation/projects-initiatives/clinical-validity-classification/) (Table 1; Supp. Tables S1–S14). The scores for each disease are very consistent across scorers (Intraclass correlation coefficient = 0.984), and the clinical validity category “bins” scores to increase consistency of the final result. Two main sources of variability in scoring are determining which patients can be counted for clinical validity scoring and whether functional experiments are sufficient to merit points for variant pathogenicity.

**Table 1:** Clinical Validity Scores for a Set of Gene–Disease Relationships

		Clinical validity score						
HGNC gene symbol	Disease curated	OMIM ID		Curator 1		Curator 2		Curator 3
AKAP9	Long QT syndrome	611820	5	Limited	5	Limited	5	Limited
ARSD	Chondrodysplasia punctata	–	0	No reported patients	0	No reported patients	0	No reported patients
ATF6	Achromatopsia	616517	13	Strong	14	Strong	14	Strong
C1QB	Immunodeficiency due to early C1q deficiency	613652	11	Moderate	11	Moderate	12	Moderate
CD3E	Severe combined immunodeficiency	615615	13	Strong	14	Strong	14	Strong
CHD1L	Renal or urinary tract malformation (CAKUT)		5	Limited	5	Limited	4	Limited
COL2A1	Spondyloepiphyseal dysplasia, Stanescu type	616583	11	Moderate	12	Moderate	12	Moderate
DICER1	Pleuropulmonary blastoma	601200	–	Definitive	–	Definitive	–	Definitive
FGFR3	Achondroplasia	100800	–	Definitive	–	Definitive	–	Definitive
NGLY1	Congenital disorder of deglycosylation	615273	15	Strong	15	Strong	13	Strong
NHP2	Dyskeratosis congenita	613987	8	Moderate	9	Moderate	8	Moderate
PALB2	Hereditary breast cancer	114480	–	Definitive	–	Definitive	–	Definitive
PMS2	Pancreatic cancer	–	0	No reported patients	0	No reported patients	0	No reported patients
PSD3	Antecubital pterygium syndrome	–	0	No reported patients	0	No reported patients	0	No reported patients
RAD51C	Fanconi anemia	613390	8	Moderate	8	Moderate	9	Moderate
RPS10	Diamond‐Blackfan anemia	613308	13	Strong	13	Strong	13	Strong
RPS24	Diamond‐Blackfan anemia	610629	13	Strong	13	Strong	13	Strong
SCN4B	Long QT syndrome	611819	5	Limited	7	Limited	5	Limited
SKI	Shprintzen‐Goldberg syndrome	182212	–	Definitive	–	Definitive	–	Definitive
SMAD3	Loeys‐Dietz syndrome	613795	–	Definitive	–	Definitive	–	Definitive
SOS2	Noonan syndrome	616559	11	Moderate	10	Moderate	11	Moderate
WRAP53	Dyskeratosis congenita	613988	7	Limited	9	Moderate	9	Moderate

Clinical validity scores for a gene–disease list show strong concordance across three independent scorers. One case of category discordance is highlighted.

The present clinical validity scoring system demonstrates concurrent validity by accurately describing the strength of gene–disease relationships from no evidence (no reported patients, e.g., *ARSD* [MIM# 300002], *PMS2* [MIM# 600259], and *PSD3* [MIM# 614440] from Table 1) to overwhelming evidence (definitive, e.g., *DICER1* [MIM# 606241], *FGFR3* [MIM# 134934], *SKI* [MIM# 164780], *SMAD3* [MIM# 603109]). An example of how the rules of the scoring system demonstrate robust discriminant validity can be seen in comparing evidence of the “limited” score obtained for *AKAP9* (MIM# 604001) versus the “moderate” score for *RAD51C* (MIM# 602774). Cursory review of evidence for a role of *AKAP9* in long QT syndrome shows five missense alterations in HGMD, and less evidence for a role of *RAD51C* in a Fanconi‐anemia‐like disorder: only one missense alteration in HGMD. As shown in Supp. Table S1, all three curators determined that evidence for a role of *AKAP9* in Long QT syndrome was only limited because three out of four reported patients did not meet diagnostic criteria for Long QT syndrome, eliminating them from consideration in scoring. In contrast, *RAD51C* had moderate evidence for a role in Fanconi anemia due to extensive experimental evidence: patient fibroblast proliferation assays and rescue experiments in vitro plus an animal model all supported the gene–disease relationship (Supp. Table S9). It may seem only logical to take all of these lines of evidence into consideration, but the discriminant validity of the scoring system is shown in that all curators were able to weigh the evidence using the same scale and come to similar determinations for *AKAP9* and *RAD51C*.

We have tried to define inclusion criteria as carefully as possible, and will also reference standard diagnostic criteria for each disease when available. In rare cases, discordant scores are unavoidable, however. For instance, in evaluating *WRAP53* and Dyskeratosis Congenita (Table 1; Supp. Table S14), scorers disagreed whether published functional experimental results were sufficient evidence of variant pathogenicity. The resulting scores are not substantially different, but are perhaps best interpreted as a reminder to be cautious when clinically interpreting a finding in a gene on the border of two categories. Additionally, we usually group diseases that appear to be on a phenotypic spectrum and share a mutational mechanism without apparent genotype–phenotype associations. Therefore, we scored *COL2A1* (MIM# 120140) for Stanescu‐type spondyloepiphyseal dysplasia for the sake of comparison with ClinGen's scoring set, but in daily use would likely score the collagenopathies together and simply note the variable phenotype, due to consistent mutational mechanism and overlapping features reported. Overall, across many performance measures in the absence of a “gold standard,” the present scoring system proves useful in translating the concept of clinical validity into an actionable system.

### Characterized Genes According to the Clinical Validity Scoring System

In practice, clinical validity scoring has augmented the analysis of DES most critically in how we assess gene–disease relationships at the boundary between limited and moderate evidence. Ideally, a gene–disease relationship with moderate evidence has some information about mutational mechanism, plus sufficient information on human phenotypes to inform clinical assessments. This allows a patient with a newly discovered pathogenic alteration(s) in that gene to receive a diagnosis and extrapolate from other patients’ experience to predict a disease course. This information, in a practical sense, allows the gene–disease relationship to be considered in the first level of analysis and for proband‐only cases. These results are also reported out with a higher confidence in accordance with ACMG guidelines (e.g., without at least moderate clinical significance, all findings are reported out as uncertain.). Gene–disease relationships with a score of limited or below are considered candidate genetic etiologies [Farwell Hagman et al., 2016], as they lack sufficient evidence for a definitive clinical diagnosis. Cases are only reviewed for candidate genetic diagnoses if a characterized diagnosis was not identified and if there is an informative trio to aid bioinformatics filtering—therefore, about 60% of all cases are reviewed for candidate genetic etiologies. After the first patient is published, the gene–disease relationship may no longer be considered “novel,” but is still usually scored as limited and the gene is considered a candidate until it achieves the necessary level of confirmation required to inform clinical diagnostics.

Standardized clinical validity scoring criteria can be useful not only for diagnostic exome interpretation but also for selecting genes for disease‐specific panels with the goal of maximizing analytic sensitivity and specificity. Finding genes to add to a panel is not difficult: sometimes consensus guidelines will even recommend which genes should be included on a disease‐specific gene panel. Additionally, querying our internally managed database of curated gene–disease associations has suggested more genes. The adoption of a clinical validity scoring system at our laboratory, however, has streamlined the gene content vetting process in panel creation, that is, narrowing the gene list to good candidates. Genes being considered for inclusion on a panel can be ranked by clinical validity score, and ideally only characterized genes should be included on a panel as recommended by both the European Society of Human Genetics [Matthijs et al., 2016] and the American College of Medical Genetics and Genomics [Rehm, 2013]. Including only characterized genes on panels helps reduce the number of variants of unknown significance that will be detected and ensures that patient results will be interpretable. While characterization should be necessary for a gene to be included on a panel, it may not be sufficient to support inclusion on a panel. Additional information, such as the likelihood of detecting false positives in that gene, may also come into consideration. For instance, in a few genes such as *PACS1* (MIM# 607492), only a single recurrent pathogenic variant has been reported, and therefore only alterations at that site can truly be considered characterized. How to categorize other rare alterations found in such genes can vary between laboratories and between diagnostic method, for example, DES or panel detection.

The rapid pace of discovery and intensive curation of our gene database leads us to re‐evaluate gene–disease associations often: a team of scientists review literature for new discoveries daily. When comprehensive clinical validity assessment results in the identification of the first characterized etiology for that gene, all previously reported patients with rare variants in that gene are reassessed. Review of these previous patients can sometimes allow a new diagnosis for a patient who previously received a negative report. This prompts us to initiate reanalysis and reclassification as a standard component of DES. To quantify the contribution of reclassification to DES rates, we examined our patient database.

### Rates of Reclassification

**Figure 2:**
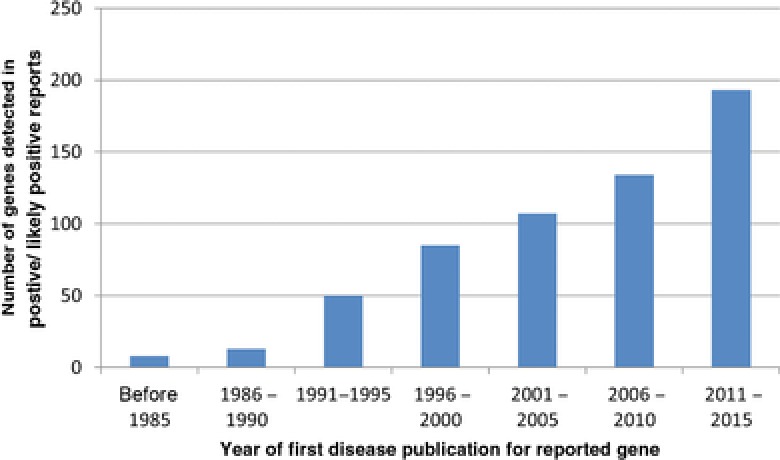
Positive reports are often recently discovered genetic etiologies. New gene–disease discoveries are the primary source of positive findings, with 32% of all positive reports reported in genetic etiologies that were discovered after 2010.

**Figure 3:**
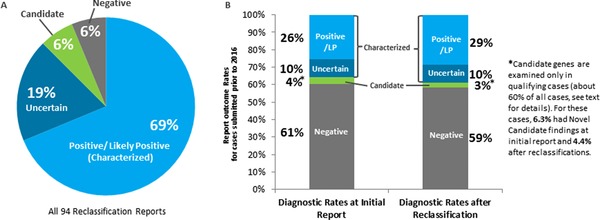
Reanalysis and reclassification leads to increased diagnostic yield. **A**: Of the 96 reclassification reports studied here, 69% identified as positive/likely positive result in a characterized gene. **B**: Diagnostic rates at initial report are 25.6% positive/likely positive in characterized genes. After at least 1 year has passed, diagnostic rates have increased to 28.6% positive/likely positive in characterized genes due to reclassification events.

In the current cohort of the first 2,112 sequential cases submitted to Ambry Genetics for testing prior to 2016, 32% of positive characterized genetic etiologies were in genes discovered since 2010 (Fig. 2). To properly determine the effect of updated information on patient reclassification, only cases that were received at least 12 months ago were analyzed, allowing time for new information to accrue. The overall results of exome analysis were changed in 96 reclassification reports, excluding reclassifications due to family studies or NGS pipeline upgrades. Overall, among all reclassification reports, 73% were reclassified due to reports of a new gene–disease discovery in medical literature. Of the reclassified cases, 69% were reclassified to positive/likely positive for a characterized gene (Fig. 3A), 19% were reclassified to uncertain, and 6% were reclassified to candidate in an uncharacterized Mendelian disease gene, as shown in Table 2. Of all reclassifications, 6% of cases were reclassified to negative.

**Table 2:** Category of Reclassification Reports for First 4 Years of Diagnostic Exome Sequencing

		Final category after reclassification report			
		Characterized		Uncharacterized	
Initial report		Positive/LP	Uncertain	Candidate	Negative
Characterized	Positive/LP	4 (4%)	1 (1%)	–	1 (1%)
	Uncertain	9 (9%)	–	–	5 (6%)
Uncharacterized	Candidate	26 (27%)	2 (2%)	–	–
	Negative	27 (28%)	15 (16%)	6 (6%)	–
	Sum	64 (69%)	18 (19%)	6 (6%)	6 (6%)

The initial report category is described in rows, whereas columns show the outcome of the reclassification report. The majority of reclassification reports are from candidate or negative initial reports switching to positive or likely positive.

Of cases that received a negative reclassification report, the majority (five) were due to updated population frequencies from control databases [Lek et al., 2016]. The remaining negative reclassification report was due to new literature disputing the pathogenicity of alterations in the identified gene (*SRI*; MIM# 182520). Within the first 500 cases, we previously reported that in cases in which a candidate genetic etiology was initially reported, 51.9% had subsequent corroborating evidence in the literature [Farwell Hagman et al., 2016]. Of the current cohort of 80 candidate genetic etiology reports, 28 subsequently were issued a reclassification report, that is, 35% of all candidate genetic etiology reports were subsequently reclassified to positive/likely positive in characterized genes. Overall, about 5.6% of cases that initially received negative or candidate results were upgraded to positive/likely positive or uncertain in a characterized gene. This statistic is expected to increase over time as new information is published, and it emphasizes the importance of timely and thorough literature review.

### Case Report: Reanalysis and Reclassification

Reclassification reports are becoming a standard‐of‐care as new information illuminates molecular diagnoses in patients who previously received a negative report. Currently, however, there is no industry‐wide consensus as to how often it is appropriate to reanalyze previously negative cases. We propose that diagnostic laboratories should review all previous cases with rare variants detected in the gene for which new evidence of at least moderate clinical validity is attained, and report all new diagnoses to the client. This systematic gene‐based approach to previous case review is likely more time‐efficient and thorough in identifying new diagnoses than individual case‐based reanalysis upon request.

As an example, our laboratory had a case in 2013 with a complex phenotype: a 7‐year‐old girl with recurrent hepatopathy with elevated transaminases, global developmental delay, poor growth, chronic anemia, skeletal dysplasia, abnormal MRI signal in the pons, and an abnormal gait. The patient's parents are first cousins, suggesting a possible recessive genetic etiology. The analysis of characterized genetic etiologies based on patient's exome data was requested and none of the 143 alterations in 124 characterized genes detected by DES showed sufficient symptom overlap to explain the phenotype, so the patient received a negative report. A patient‐requested reanalysis in July 2015 was also negative, despite new symptoms of diffuse osteopenia, beaking in the thoracolumbar spine with disc calcifications, and multiple epiphyses in the hands and feet.

Finally, an article by Schmidt et al. (2015) reported three patients from two families who presented with recurrent liver failure in early infancy, cerebellar vermis atrophy, ataxia, and peripheral neuropathy. Each patient had compound heterozygous truncating pathogenic variants in *SCYL1* (MIM# 607982). The clinical validity score for the association between *SCYL1* and spinocerebellar ataxia with hepatopathy was determined as follows: there were two unrelated affected individuals (1 point), one publication reporting patients (1 point), and all four reported variants are predicted pathogenic: two frameshift, one nonsense, and one splicing variant (4 points). The protein encoded by mouse *Scyl1* is expressed ubiquitously but localizes to synapses in neurons and at neuromuscular junctions, and where it likely contributes to retrograde protein trafficking and nuclear tRNA export (1 point for gene function) [Chafe and Mangroo, 2010; Schmidt et al., 2015]. Additionally, the authors showed that patient skin fibroblasts completely lacked the SCYL1 protein, and the Golgi apparatus is greatly enlarged in HeLa cells lacking *SCYL1* (1 point for gene disruption) [Schmidt et al., 2015]. The spontaneous animal model, the *mdf* mouse, was previously reported to have biallelic disruption of *Scyl1* and neurogenic muscular atrophy, ataxia, cerebellar atrophy, and optic‐nerve thinning (2 points for model organism). Importantly, the mouse model does not have the liver dysfunction reported in human patients, suggesting only a partial phenotype match. In total, the gene–disease association scored 10 points, garnering a clinical validity score of moderate. This new information provided the first characterized etiology for *SCYL1* in our database.

The patient had a homozygous c.1039C>T (p.Q347^*^) alteration in the *SCYL1* gene and a significant phenotypic overlap with the published patients. She was therefore issued a reclassification report in December 2015 with this new diagnosis. This diagnosis confirms the association between biallelic loss‐of‐function alterations in *SCYL1* and a syndrome of recurrent liver failure with ataxia. Whether this patient's skeletal symptoms are an expansion of phenotype or are due to other comorbid pathogenic variants can be distinguished as more patients are reported. In follow‐up communication, the patient's physician agreed that the patient fits very well with the described phenotype. Importantly, the patient recently started to complain of pain and swelling in her hands and feet. The physician recognized that this could be the development of peripheral neuropathy described in the published patients [Schmidt et al., 2015], and referred the patient to neurology for further care.

This patient case demonstrates the importance of timely literature reviews, standardized assessment of clinical validity, and producing the associated patient reclassification reports. With complete phenotypic information due to diagnosis of a characterized gene, physicians are better able to monitor and treat patients. For some diseases, exome sequencing can even contribute to finding intervention: recently, DES in a cohort of patients with intellectual developmental disorder and unexplained metabolic abnormalities was reported to lead to a change in treatment in 44% of cases [Tarailo‐Graovac et al., 2016].

## Discussion

A well‐curated characterized gene database promotes accurate phenotype‐driven exome analysis. It enables reclassification as new studies are published, providing patients with a diagnosis and opportunities for therapeutic options, and an end to their “diagnostic odyssey.” There is a distinct advantage of trio sequencing for the discovery of candidate genetic etiologies; the resultant well‐defined gene/alteration list after trio filtering enables a thorough literature search of each prioritized gene, minimizing the chance of missing a positive finding at a newly discovered locus and revealing the origin of the potential pathogenic or likely pathogenic alteration in a new etiology in reanalysis. In autosomal‐dominant diseases, the detection of a pathogenic de novo alteration in an affected proband with unaffected parents can guide diagnosis. For instance, the first *GNAO1* (MIM# 139311) positive case at our laboratory was identified on exactly the same day that the gene was published as a Mendelian disease gene in the American Journal of Human Genetics [Nakamura et al., 2013]. Over time and with consistent literature review and clinical validity curation, we expect that many patients with candidate genetic etiologies will receive a definitive diagnosis via reclassification reports. Furthermore, maintaining an up‐to‐date database of these newly characterized genes can also aid patients seeking diagnosis via gene panels or DES without an informative trio.

The main drawbacks of this scoring system have to do with the requirement for diseases to be Mendelian, highly penetrant, extremely rare, and specifically defined to be scored. It is much more difficult to accurately assess the weight of evidence for a role of a gene in multigenic diseases than in monogenic diseases. Perhaps some adaptations to the scoresheet can accommodate these complex gene–disease relationships. Additionally, assessing new gene–disease relationships, especially for relatively common disorders such as autism, is inherently complicated by appropriately defining the disease to score, as illustrated by many of the newly published neurodevelopmental genes. Diagnosis for patients with pathogenic alterations in such genes will greatly depend on molecular findings due to the nonspecific presentations and high variability of common features. The ascertainment of clinical validity for these genes relies heavily on statistical significance from large cohort studies. For diseases with variable syndromic phenotypes, it can be challenging to determine which patients have separate diseases and therefore which count toward each disease score. In particular, candidate genetic etiologies lack published reports of the full clinical spectrum of disease. Until the full clinical spectrum of a disease is published, it may be prudent to score patients with different symptoms as having separate diseases. For instance, *SNAP25* (MIM# 600322) currently has scores of limited for an epilepsy phenotype [Rohena et al., 2013] and an ataxia‐contractures phenotype [Shen et al., 2014], so *SNAP25* is still a candidate gene. The recent conference presentation of a patient with both major phenotypes broadens the clinical spectrum and will allow the gene *SNAP25* to be considered characterized once published [Gabriel et al., 2016].

Monitoring newly published discoveries and reviewing previous cases can allow genetics laboratories to retrospectively diagnose patients. Whenever a gene–disease association first achieves a score of moderate or above, all patients with rare alterations in that gene are reviewed manually for phenotype–genotype fit. While computer‐assisted review of phenotypic terminology may reduce the time and effort required in this process, it might miss cases of phenotype expansion, as suggested by statistical analysis of a large cohort for new disease gene discovery [McRae et al., 2017]. On average, during 2016, about two genes have switched to characterized per week, necessitating review of all patients with rare variants previously detected in each of these genes. Any finding that leads to a new diagnosis is sent to ordering physicians in a reclassification report. The process of recontacting patients via ordering physicians to provide the results can be quite burdensome [Carrieri et al., 2016]. Furthermore, there are no industry guidelines regarding reimbursement for reanalysis or how often it should be performed. Clinical validity assessment, systematic reanalysis of past reports, and writing and issuing reclassification reports is a significant financial burden for diagnostic laboratories. However, given the rapidly advancing gene–disease data, we believe that it is essential to factor these costs into the overall test cost in order to ensure optimal patient care.

## Future Directions

Our experience highlights the importance of careful literature curation and evaluation using a system of clinical validity scoring optimized for use in a diagnostic laboratory. This scoring system is simple enough to be quickly implemented while updating a gene database with the latest findings, and it can specifically guide reporting decisions at the important boundary of limited and moderate evidence, determining whether a gene is characterized. Further, we find that review of previous cases while updating clinical validity of gene–disease relationships can contribute to improved patient care, and reclassification reports can increase diagnostic rate. The rapid pace of discovery in genetic diagnostics emphasizes a need for data sharing. A database similar to ClinVar, in which laboratories can submit clinical validity scores for curated genes, would be a useful tool for consistent diagnostic reporting across laboratories. In the meantime, our working categorization of genes as characterized or novel candidates will be available at AmbryShare (https://share.ambrygen.com/). The importance of data sharing has been demonstrated repeatedly during the development of DES: patients stand to gain a diagnosis informed by current knowledge in the field, and medical knowledge can increase from the combined findings of researchers all over the world.

E.D.S., K.R., M.R., D.N.S., S.D., D.E.K., Z.P., K.L.H., K.W., S.T., and K.D.F.H. are employed by and receive a salary from Ambry Genetics. Exome sequencing is among the commercially available tests at Ambry Genetics.

## Supporting information

Supplementary Table S1.
Supplementary Table S2.
Supplementary Table S3.
Supplementary Table S4.
Supplementary Table S5.
Supplementary Table S6.
Supplementary Table S7.
Supplementary Table S8.
Supplementary Table S9.
Supplementary Table S10.
Supplementary Table S11.
Supplementary Table S12.
Supplementary Table S13.
Supplementary Table S14.

## References

[humu23183-bib-0001] Amendola LM , Jarvik GP , Leo MC , McLaughlin HM , Akkari Y , Amaral MD , Berg JS , Biswas S , Bowling KM , Conlin LK , Cooper GM , Dorschner MO , et al. 2016 Performance of ACMG‐AMP variant‐interpretation guidelines among nine laboratories in the clinical sequencing exploratory research consortium. Am J Hum Genet 98:1067–1076. 27181684 10.1016/j.ajhg.2016.03.024 PMC4908185

[humu23183-bib-0002] Biesecker LG , Green RC . 2014 Diagnostic clinical genome and exome sequencing. N Engl J Med 371:1170. 10.1056/NEJMc1408914 25229935

[humu23183-bib-0003] Carrieri D , Lucassen AM , Clarke AJ , Dheensa S , Doheny S , Turnpenny PD , Kelly SE . 2016 Recontact in clinical practice: a survey of clinical genetics services in the United Kingdom. Genet Med 18:876–881. 26890453 10.1038/gim.2015.194 PMC5052431

[humu23183-bib-0004] Chafe SC , Mangroo D . 2010 Scyl1 facilitates nuclear tRNA export in mammalian cells by acting at the nuclear pore complex. Mol Biol Cell 21:2483–2499. 20505071 10.1091/mbc.E10-03-0176 PMC2903676

[humu23183-bib-0005] Chong JX , Buckingham KJ , Jhangiani SN , Boehm C , Sobreira N , Smith JD , Harrell TM , McMillin MJ , Wiszniewski W , Gambin T , Coban Akdemir ZH , Doheny K , et al. 2015 The genetic basis of Mendelian phenotypes: discoveries, challenges, and opportunities. Am J Hum Genet 97:199–215. 26166479 10.1016/j.ajhg.2015.06.009 PMC4573249

[humu23183-bib-0006] ClinGen . 2016 Clinical validity curation spreadsheet. Clinical Genome Resource https://www.clinicalgenome.org/working-groups/gene-curation/projects-initiatives/gene-disease-clinical-validity-scoring-matrix/.

[humu23183-bib-0007] Cooper DN , Chen JM , Ball EV , Howells K , Mort M , Phillips AD , Chuzhanova N , Krawczak M , Kehrer‐Sawatzki H , Stenson PD . 2010 Genes, mutations, and human inherited disease at the dawn of the age of personalized genomics. Hum Mutat 31:631–655. 20506564 10.1002/humu.21260

[humu23183-bib-0018] Deciphering Developmental Disorders S . 2017 Prevalence and architecture of de novo mutations in developmental disorders. Nature. 10.1038/nature21062 PMC6016744 28135719

[humu23183-bib-0008] Farwell Hagman KD , Shinde DN , Mroske C , Smith E , Radtke K , Shahmirzadi L , El‐Khechen D , Powis Z , Chao EC , Alcaraz WA , Helbig KL , Sajan SA , et al. 2016 Candidate‐gene criteria for clinical reporting: diagnostic exome sequencing identifies altered candidate genes among 8% of patients with undiagnosed diseases. Genet Med. 2016 Aug 11. doi:10.1038/gim.2016.95. [Epub ahead of print] 10.1038/gim.2016.95 PMC5303763 27513193

[humu23183-bib-0009] Farwell KD , Shahmirzadi L , El‐Khechen D , Powis Z , Chao EC , Tippin Davis B , Baxter RM , Zeng W , Mroske C , Parra MC , Gandomi SK , Lu I , et al. 2015 Enhanced utility of family‐centered diagnostic exome sequencing with inheritance model‐based analysis: results from 500 unselected families with undiagnosed genetic conditions. Genet Med 17:578–586. 25356970 10.1038/gim.2014.154

[humu23183-bib-0010] Gabriel H FS , Stoebe P , Hoertnagel K , Biskup S , Schell‐Apacik C . 2016. A de novo mutation in the SNAP25 gene in a patient with epileptic encephalopathy, hypotonia and contractures identified by trio‐based exome sequencing. Barcelona, Spain: CCIB https://www.eshg.org/fileadmin/www.eshg.org/conferences/2016/downloads/ESHG2016_Abstracts_final.pdf.

[humu23183-bib-0011] Kamien B , Lionel AC , Bain N , Scherer SW , Hunter M . 2014 Outfoxed by RBFOX1—a caution about ascertainment bias. Am J Med Genet A 164a:1411–1418. 24664471 10.1002/ajmg.a.36458

[humu23183-bib-0012] Laduca H , Stuenkel AJ , Dolinsky JS , Keiles S , Tandy S , Pesaran T , Chen E , Gau CL , Palmaer E , Shoaepour K , Shah D , Speare V , Gandomi S , Chao E . 2014 Utilization of multigene panels in hereditary cancer predisposition testing: analysis of more than 2,000 patients. Genet Med 16:830–837. 10.1038/gim.2014.40 PMC4225457 24763289

[humu23183-bib-0013] Lazaridis KN , Schahl KA , Cousin MA , Babovic‐Vuksanovic D , Riegert‐Johnson DL , Gavrilova RH , McAllister TM , Lindor NM , Abraham RS , Ackerman MJ , Pichurin PN , Deyle DR , et al. 2016 Outcome of whole exome sequencing for diagnostic odyssey cases of an individualized medicine clinic: the mayo clinic experience. Mayo Clin Proc 91:297–307. 26944241 10.1016/j.mayocp.2015.12.018

[humu23183-bib-0014] Lee H , Deignan JL , Dorrani N , Strom SP , Kantarci S , Quintero‐Rivera F , Das K , Toy T , Harry B , Yourshaw M , Fox M , Fogel BL , et al. 2014 Clinical exome sequencing for genetic identification of rare Mendelian disorders. JAMA 312:1880–1887. 25326637 10.1001/jama.2014.14604 PMC4278636

[humu23183-bib-0015] Lek M , Karczewski KJ , Minikel EV , Samocha KE , Banks E , Fennell T , O'Donnell‐Luria AH , Ware JS , Hill AJ , Cummings BB , Tukiainen T , Birnbaum DP , et al. 2016 Analysis of protein‐coding genetic variation in 60,706 humans. Nature 536:285–291. 27535533 10.1038/nature19057 PMC5018207

[humu23183-bib-0016] MacArthur DG , Manolio TA , Dimmock DP , Rehm HL , Shendure J , Abecasis GR , Adams DR , Altman RB , Antonarakis SE , Ashley EA , Barett JC , Biesecker LG , et al. 2014 Guidelines for investigating causality of sequence variants in human disease. Nature 508:469–476. 24759409 10.1038/nature13127 PMC4180223

[humu23183-bib-0017] Matthijs G , Souche E , Alders M , Corveleyn A , Eck S , Feenstra I , Race V , Sistermans E , Sturm M , Weiss M , Yntema H , Bakker E , et al. 2016 Guidelines for diagnostic next‐generation sequencing. Eur J Hum Genet 24:2–5. 26508566 10.1038/ejhg.2015.226 PMC4795226

[humu23183-bib-0019] Medicine M‐NIoG . 1966–2016 Online Mendelian inheritance in man, OMIM^®^. Baltimore, MD: Johns Hopkins University.

[humu23183-bib-0020] Nakamura K , Kodera H , Akita T , Shiina M , Kato M , Hoshino H , Terashima H , Osaka H , Nakamura S , Tohyama J , Kumada T , Furukawa T , et al. 2013 De novo mutations in GNAO1, encoding a Galphao subunit of heterotrimeric G proteins, cause epileptic encephalopathy. Am J Hum Genet 93:496–505. 23993195 10.1016/j.ajhg.2013.07.014 PMC3769919

[humu23183-bib-0021] National Human Genome Research I . 2013 Quantitative advances since the human genome project. World Wide Web https://www.genome.gov/images/illustrations/hgp_measures.pdf.

[humu23183-bib-0022] Pesaran T , Karam R , Huether R , Li S , Farber‐Katz S , Chamberlin A , Chong H , LaDuca H , Elliott A . 2016 Beyond DNA: an integrated and functional approach for classifying germline variants in breast cancer genes. Int J Breast Cancer 2016:2469523. 27822389 10.1155/2016/2469523 PMC5086358

[humu23183-bib-0023] Piton A , Redin C , Mandel JL . 2013 XLID‐causing mutations and associated genes challenged in light of data from large‐scale human exome sequencing. Am J Hum Genet 93:368–383. 23871722 10.1016/j.ajhg.2013.06.013 PMC3738825

[humu23183-bib-0024] Rehm HL . 2013 Disease‐targeted sequencing: a cornerstone in the clinic. Nat Rev Genet 14:295–300. 23478348 10.1038/nrg3463 PMC3786217

[humu23183-bib-0025] Retterer K , Juusola J , Cho MT , Vitazka P , Millan F , Gibellini F , Vertino‐Bell A , Smaoui N , Neidich J , Monaghan KG , McKnight D , Bai R , et al. 2015 Clinical application of whole‐exome sequencing across clinical indications. Genet Med 18:696–704. 26633542 10.1038/gim.2015.148

[humu23183-bib-0026] Richards S , Aziz N , Bale S , Bick D , Das S , Gastier‐Foster J , Grody WW , Hegde M , Lyon E , Spector E , Voelkerding K , Rehm HL , et al. 2015 Standards and guidelines for the interpretation of sequence variants: a joint consensus recommendation of the American college of medical genetics and genomics and the association for molecular pathology. Genet Med 17:405–424. 25741868 10.1038/gim.2015.30 PMC4544753

[humu23183-bib-0027] Rohena L , Neidich J , Truitt Cho M , Gonzalez KD , Tang S , Devinsky O , Chung WK . 2013 Mutation in SNAP25 as a novel genetic cause of epilepsy and intellectual disability. Rare Dis 1:e26314. 25003006 10.4161/rdis.26314 PMC3932847

[humu23183-bib-0028] Samocha KE , Robinson EB , Sanders SJ , Stevens C , Sabo A , McGrath LM , Kosmicki JA , Rehnstrom K , Mallick S , Kirby A , Wall DP , MacArthur DG , et al. 2014 A framework for the interpretation of de novo mutation in human disease. Nat Genet 46:944–950. 25086666 10.1038/ng.3050 PMC4222185

[humu23183-bib-0029] Schmidt WM , Rutledge SL , Schule R , Mayerhofer B , Zuchner S , Boltshauser E , Bittner RE . 2015 Disruptive SCYL1 mutations underlie a syndrome characterized by recurrent episodes of liver failure, peripheral neuropathy, cerebellar atrophy, and ataxia. Am J Hum Genet 97:855–861. 26581903 10.1016/j.ajhg.2015.10.011 PMC4678415

[humu23183-bib-0030] Shen XM , Selcen D , Brengman J , Engel AG . 2014 Mutant SNAP25B causes myasthenia, cortical hyperexcitability, ataxia, and intellectual disability. Neurology 83:2247–2255. 25381298 10.1212/WNL.0000000000001079 PMC4277673

[humu23183-bib-0031] Soden SE , Saunders CJ , Willig LK , Farrow EG , Smith LD , Petrikin JE , LePichon JB , Miller NA , Thiffault I , Dinwiddie DL , Twist G, Noll A, et al. 2014 Effectiveness of exome and genome sequencing guided by acuity of illness for diagnosis of neurodevelopmental disorders. Sci Transl Med 6:265ra168. 10.1126/scitranslmed.3010076 PMC4286868 25473036

[humu23183-bib-0032] Srivastava S , Cohen JS , Vernon H , Baranano K , McClellan R , Jamal L , Naidu S , Fatemi A . 2014 Clinical whole exome sequencing in child neurology practice. Ann Neurol 76:473–483. 25131622 10.1002/ana.24251

[humu23183-bib-0033] Stenson PD , Mort M , Ball EV , Shaw K , Phillips A , Cooper DN . 2014 The Human Gene Mutation Database: building a comprehensive mutation repository for clinical and molecular genetics, diagnostic testing and personalized genomic medicine. Hum Genet 133:1–9. 24077912 10.1007/s00439-013-1358-4 PMC3898141

[humu23183-bib-0034] Tarailo‐Graovac M , Shyr C , Ross CJ , Horvath GA , Salvarinova R , Ye XC , Zhang LH , Bhavsar AP , Lee JJ , Drogemoller BI , Abdelsayed M , Alfadhel M , et al. 2016 Exome sequencing and the management of neurometabolic disorders. N Engl J Med 374:2246–2255. 27276562 10.1056/NEJMoa1515792 PMC4983272

[humu23183-bib-0035] Yang Y , Muzny DM , Reid JG , Bainbridge MN , Willis A , Ward PA , Braxton A , Beuten J , Xia F , Niu Z , Hardison M , Person R , et al. 2013 Clinical whole‐exome sequencing for the diagnosis of Mendelian disorders. N Engl J Med 369:1502–1511. 24088041 10.1056/NEJMoa1306555 PMC4211433

[humu23183-bib-0036] Yang Y , Muzny DM , Xia F , Niu Z , Person R , Ding Y , Ward P , Braxton A , Wang M , Buhay C , Veeraraghavan N , Hawes A , et al. 2014 Molecular findings among patients referred for clinical whole‐exome sequencing. JAMA 312:1870–1879. 25326635 10.1001/jama.2014.14601 PMC4326249

