# Supplementary material for: Classification of Genes: Standardized Clinical Validity Assessment of Gene–Disease Associations Aids Diagnostic Exome Analysis and Reclassifications
Source: Hum Mutat. 2017 Feb 13;38(5):600–8. doi: 10.1002/humu.23183 (PMC5655771; doi:10.1002/humu.23183)
Supplement: Supplementary file 1 — Supplementary Table S1. Supplementary Table S2. Supplementary Table S3. Supplementary Table S4. Supplementary Table S5. Supplementary Table S6. Supplementary Table S7. Supplementary Table S8. Supplementary Table S9. Supplementary Table S10. Supplementary Table S11. Supplementary Table S12. Supplementary Table S13. Supplementary Table S14. [file HUMU-38-600-s001.pdf]

**Supplementary Table S1. *AKAP9* and Long QT/ Romano-Ward Syndrome**				
	Curator 1	Curator 2	Curator 3	References
Genetic Evidence				
# Probands (0-4)	1	1	1	(Chen, Marquardt et al. 2007)
# Publications (0-3)	1	1	1	
# Variants (0-4)	1	1	1	
Other Statistical Evidence (0-1)				
Experimental Evidence				
Gene Function (0-2)	2	2	2	(Chen, Marquardt et al. 2007, Mullally, Goldenberg et al. 2013, Brion, Blanco-Verea et al. 2014, Shigemizu, Aiba et al. 2015)
Gene Disruption (0-2)				
Animal Model (0-2)				
Calculated score	5	5	5	
Curator Classification	Limited	Limited	Limited	

Notes:
Mullally *et al*. 2013 reported a patient with c.2113T>G p.L705V detected in *AKAP9* from a screen of Long QT genes, but this alteration was detected possibly in combination with alterations in other genes, so omit from consideration.
Brion *et al.* 2014 reported a patient with c.A9943G p.T3315A and LQTS within a mixed cohort with no specific patient phenotype, so do not count.
Shigemezu *et al.* 2015 reported 2 patients with c.5341T>A p.S1781T and c.2295T>A p.D765E, but asymptomatic and QTc is within normal range, so do not count.

Supplementary Table S2. *ATF6* and Achromatopsia				
	Curator 1	Curator 2	Curator 3	References
Genetic Evidence				
# Probands (0-4)	4	4	4	(Ansar, Santos-Cortez et al. 2015, Kohl, Zobor et al. 2015, Xu, Gelowani et al. 2015)
# Publications (0-3)	3	3	3	
# Variants (0-4)	3	3	3	
Other Statistical Evidence (0-1)		1	1	(Kohl, Zobor et al. 2015)
Experimental Evidence				
Gene Function (0-2)	1	1	1	(Ansar, Santos-Cortez et al. 2015)
Gene Disruption (0-2)				
Animal Model (0-2)	2	2	2	(Nakanishi, Shimazawa et al. 2013, Kohl, Zobor et al. 2015)
Calculated score	13	14	14	
Curator Classification	Strong	Strong	Strong	

**Supplementary Table S3.** ***C1QB*** **and Immunodeficiency due to Early C1q Deficiency**				
	Curator 1	Curator 2	Curator 3	References
Genetic Evidence				
# Probands (0-4)	3	3	3	(McAdam, Goundis et al. 1988, Petry, Hauptmann et al. 1997, Marquart, Schejbel et al. 2007, Roumenina, Sene et al. 2011, Higuchi, Shimizu et al. 2013, Troedson, Wong et al. 2013, van Schaarenburg, Daha et al. 2015)
# Publications (0-3)	3	3	3	
# Variants (0-4)	3	3	3	
Other Statistical Evidence (0-1)				
Experimental Evidence				
Gene Function (0-2)	2	2	2	(Higuchi, Shimizu et al. 2013)
Gene Disruption (0-2)			1	(van Schaarenburg, Daha et al. 2015)
Animal Model (0-2)				(Miura-Shimura, Nakamura et al. 2002)
Other				
Calculated score	11	11	12	
Curator Classification	Moderate	Moderate	Moderate	

Notes:
The precise location of the genetic alteration in the Miura-Shimura mouse is not clear, so do not count.

Supplementary Table S4. *CD3E* and Severe Combined Immunodeficiency				
	Curator 1	Curator 2	Curator 3	References
Genetic Evidence				
# Probands (0-4)	2	2	2	(Soudais, de Villartay et al. 1993, de Saint Basile, Geissmann et al. 2004, Fuehrer, Pannicke et al. 2014)
# Publications (0-3)	3	4	3	
# Variants (0-4)	3	3	4	
Other Statistical Evidence (0-1)				
Experimental Evidence				
Gene Function (0-2)	2	2	2	(DeJarnette, Sommers et al. 1998)
Gene Disruption (0-2)	2	1	1	(Renard, Ardouin et al. 1995, DeJarnette, Sommers et al. 1998)
Animal Model (0-2)	1	2	2	(DeJarnette, Sommers et al. 1998)
Calculated score	13	14	14	
Curator Classification	Strong	Strong	Strong	

Supplementary Table S5. *CHD1L* and Renal or Urinary Tract malformation (CAKUT)				
	Curator 1	Curator 2	Curator 3	References
Genetic Evidence				
# Probands (0-4)	2	2	1	(Brockschmidt, Chung et al. 2012, Hwang, Dworschak et al. 2014, Nicolaou, Pulit et al. 2016)
# Publications (0-3)	2	2	1	
# Variants (0-4)				
Other Statistical Evidence (0-1)				
Experimental Evidence				
Gene Function (0-2)	1	1		(Ahel, Horejsi et al. 2009)
Gene Disruption (0-2)				
Animal Model (0-2)				
Calculated score	5	5	4	
Curator Classification	Limited	Limited	Limited	

Notes:
Hwang et al. 2014 reported 5 families with CAKUT and five separate missense alterations in CDH1L; only 2 of the families had rare enough variants to be considered: c.998C>G p.P333R is predicted probably damaging/deleterious and not found in ExAC and c.1199A>G p.E400G is predicted probably damaging/deleterious and found in 2 hets in ExAC: VUS.
Brockschmidt et al. 2012: reported three patients with het alterations in CDH1L plus functional studies; transfected HEK293T cells with c-myc-tagged wild-type and mutant CHD1L constructs showed interaction between PARP1 and WT CHD1L protein but not mutant CHD1L protein. All three alterations are too common in controls (ExAC) to cause disease, omit entire paper from consideration.
Nicolaou et al. 2016 was a study representing the largest set of genes analyzed in CAKUT patients to date, the contribution of previously implicated genes to CAKUT risk was significantly smaller than expected, and the disease may be more complex than previously assumed. Only c.1291_1293delACA from Nicolaou (2015) is rare enough to cause disease and predicted pathogenic. The authors report 2 patients with c.1386-2A>G, but one also had a variant in UMOD, plus this variant is found in 109 hets in ExAC, so omit from consideration.

**Supplementary Table S6. *COL2A1* and Spondyloepiphyseal Dysplasia, Stanescu Type**				
	Curator 1	Curator 2	Curator 3	References
Genetic Evidence				
# Probands (0-4)	2	2	2	(Rukavina, Mortier et al. 2014, Jurgens, Sobreira et al. 2015, Hammarsjo, Nordgren et al. 2016)
# Publications (0-3)	3	3	3	
# Variants (0-4)	3	3	3	
Other Statistical Evidence (0-1)				
Experimental Evidence				
Gene Function (0-2)	2	1	1	(Jensen, Steplewski et al. 2011, Arita, Fertala et al. 2015)
Gene Disruption (0-2)		1	1	(Jensen, Steplewski et al. 2011)
Animal Model (0-2)	1	2	2	(Li, Prockop et al. 1995, Arita, Fertala et al. 2015)
Calculated score	11	12	12	
Curator Classification	Moderate	Moderate	Moderate	

**Supplementary Table S7. *NGLY1* and Congenital Disorder of Deglycosylation**				
	Curator 1	Curator 2	Curator 3	References
Genetic Evidence				
# Probands (0-4)	3	4	4	(Need, Shashi et al. 2012, Enns, Shashi et al. 2014, Caglayan, Comu et al. 2015, He, Grotzke et al. 2015, Heeley and Shinawi 2015, Karaca, Harel et al. 2015, Bosch, Boonstra et al. 2016)
# Publications (0-3)	4	3	4	
# Variants (0-4)	4	4	3	
Other Statistical Evidence (0-1)				
Experimental Evidence				
Gene Function (0-2)	1	1	1	(Zhou, Zhao et al. 2006, Enns, Shashi et al. 2014)
Gene Disruption (0-2)	2	2	1	(He, Grotzke et al. 2015, Huang, Harada et al. 2015)
Animal Model (0-2)	1	1		(Habibi-Babadi, Su et al. 2010)
Calculated score	15	15	13	
Curator Classification	Strong	Strong	Strong	

**Supplementary Table S8. *NHP2* and Dyskeratosis Congenita**				
	Curator 1	Curator 2	Curator 3	References
Genetic Evidence				
# Probands (0-4)	1	1	1	(Vulliamy, Beswick et al. 2008, Trahan, Martel et al. 2010)
# Publications (0-3)	1	1	1	
# Variants (0-4)	3	3	2	
Other Statistical Evidence (0-1)				
Experimental Evidence				
Gene Function (0-2)	2	2	2	(Pogacic, Dragon et al. 2000)
Gene Disruption (0-2)	1	2	2	(Vulliamy, Beswick et al. 2008)
Animal Model (0-2)				
Calculated score	8	9	8	
Curator Classification	Moderate	Moderate	Moderate	

Supplementary Table S9. ***RAD51C* and Fanconi Anemia**				
	Curator 1	Curator 2	Curator 3	References
Genetic Evidence				
# Probands (0-4)	1	1	1	(Vaz, Hanenberg et al. 2010, Somyajit, Subramanya et al. 2012)
# Publications (0-3)	1	1	1	
# Variants (0-4)	1	1	1	
Other Statistical Evidence (0-1)				
Experimental Evidence				
Gene Function (0-2)	2	2	2	(Somyajit, Subramanya et al. 2012)
Gene Disruption (0-2)	2	2	2	(Vaz, Hanenberg et al. 2010, Rodrigue, Coulombe et al. 2013)
Animal Model (0-2)	1	1	2	(Kuznetsov, Haines et al. 2009, Smeenk, de Groot et al. 2010)
Calculated score	8	8	9	
Curator Classification	Moderate	Moderate	Moderate	

Supplementary Table S10. *RPS10* and Diamond-Blackfan Anemia				
	Curator 1	Curator 2	Curator 3	References
Genetic Evidence				
# Probands (0-4)	3	3	3	(Doherty, Sheen et al. 2010, Yazaki, Kamei et al. 2012, Smetanina, Mersiyanova et al. 2015)
# Publications (0-3)	3	3	3	
# Variants (0-4)	4	4	4	
Other Statistical Evidence (0-1)				
Experimental Evidence				
Gene Function (0-2)	2	2	2	(Doherty, Sheen et al. 2010)
Gene Disruption (0-2)	1	1	1	(Doherty, Sheen et al. 2010)
Animal Model (0-2)				
Calculated score	13	13	13	
Curator Classification	Strong	Strong	Strong	

Supplementary Table S11. *RPS24* and Diamond-Blackfan Anemia				
	Curator 1	Curator 2	Curator 3	References
Genetic Evidence				
# Probands (0-4)	3	3	3	(Gazda, Grabowska et al. 2006, Badhai, Frojmark et al. 2009, Boria, Garelli et al. 2010, Quarello, Garelli et al. 2010, Ghemlas, Li et al. 2015, Smetanina, Mersiyanova et al. 2015)
# Publications (0-3)	3	3	3	
# Variants (0-4)	4	4	4	
Other Statistical Evidence (0-1)				
Experimental Evidence				
Gene Function (0-2)	2	2	2	(Choesmel, Fribourg et al. 2008)
Gene Disruption (0-2)	1	1	1	(Choesmel, Fribourg et al. 2008, Badhai, Frojmark et al. 2009)
Animal Model (0-2)				
Calculated score	13	13	13	
Curator Classification	Strong	Strong	Strong	

**Supplementary Table S12. *SCN4B* and Long QT Syndrome**				
	Curator 1	Curator 2	Curator 3	References
Genetic Evidence				
# Probands (0-4)	1	1	1	(Medeiros-Domingo, Kaku et al. 2007, Li, Wang et al. 2013)
# Publications (0-3)	3	2	2	
# Variants (0-4)			1	
Other Statistical Evidence (0-1)				
Experimental Evidence				
Gene Function (0-2)	1	2	1	(Olesen, Jespersen et al. 2011)
Gene Disruption (0-2)		2		(Medeiros-Domingo, Kaku et al. 2007)
Animal Model (0-2)				
Calculated score	5	7	5	
Curator Classification	Limited	Limited	Limited	

Notes:
Mullally (Mullally, Goldenberg et al. 2013) reported c.658G>A p.A220T in a patient with multiple mutations in long-QT associated genes, so omit from consideration.
Lieve (Lieve, Williams et al. 2013) reported an additional patient with c.658G>A p.A220T, but it’s not clear if mutations in multiple genes were identified in this patient, so do not count.

**Supplementary Table S13. *SOS2* and Noonan Syndrome**				
	Curator 1	Curator 2	Curator 3	References
Genetic Evidence				
# Probands (0-4)	3	3	3	(Cordeddu, Yin et al. 2015, Yamamoto, Aguena et al. 2015)
# Publications (0-3)	2	2	2	
# Variants (0-4)	3	3	3	
Other Statistical Evidence (0-1)				
Experimental Evidence				
Gene Function (0-2)	2	2	2	(Yamamoto, Aguena et al. 2015)
Gene Disruption (0-2)	1		1	(Cordeddu, Yin et al. 2015)
Animal Model (0-2)				
Calculated score	11	10	11	
Curator Classification	Moderate	Moderate	Moderate	

Supplementary Table S14. *WRAP53* and Dyskeratosis Congenita				
	Curator 1	Curator 2	Curator 3	References
Genetic Evidence				
# Probands (0-4)	1	1	1	(Batista, Pech et al. 2011, Zhong, Savage et al. 2011)
# Publications (0-3)	1	1	1	
# Variants (0-4)	2	4	4	
Other Statistical Evidence (0-1)				
Experimental Evidence				
Gene Function (0-2)	2	2	2	(Venteicher, Abreu et al. 2009)
Gene Disruption (0-2)	1	1	1	(Batista, Pech et al. 2011)
Animal Model (0-2)				
Calculated score	7	9	9	
Curator Classification	Limited	Moderate	Moderate	

REFERENCES

Ahel, D., Z. Horejsi, N. Wiechens, S. E. Polo, E. Garcia-Wilson, I. Ahel, H. Flynn, M. Skehel, S. C. West, S. P. Jackson, T. Owen-Hughes and S. J. Boulton (2009). "Poly(ADP-ribose)-dependent regulation of DNA repair by the chromatin remodeling enzyme ALC1." Science **325**(5945): 1240-1243.

Ansar, M., R. L. Santos-Cortez, M. A. Saqib, F. Zulfiqar, K. Lee, N. M. Ashraf, E. Ullah, X. Wang, S. Sajid, F. S. Khan, M. Amin-ud-Din, J. D. Smith, J. Shendure, M. J. Bamshad, D. A. Nickerson, A. Hameed, S. Riazuddin, Z. M. Ahmed, W. Ahmad and S. M. Leal (2015). "Mutation of ATF6 causes autosomal recessive achromatopsia." Hum Genet **134**(9): 941-950.

Arita, M., J. Fertala, C. Hou, A. Steplewski and A. Fertala (2015). "Mechanisms of aberrant organization of growth plates in conditional transgenic mouse model of spondyloepiphyseal dysplasia associated with the R992C substitution in collagen II." Am J Pathol **185**(1): 214-229.

Badhai, J., A. S. Frojmark, J. D. E, J. Schuster and N. Dahl (2009). "Ribosomal protein S19 and S24 insufficiency cause distinct cell cycle defects in Diamond-Blackfan anemia." Biochim Biophys Acta **1792**(10): 1036-1042.

Batista, L. F., M. F. Pech, F. L. Zhong, H. N. Nguyen, K. T. Xie, A. J. Zaug, S. M. Crary, J. Choi, V. Sebastiano, A. Cherry, N. Giri, M. Wernig, B. P. Alter, T. R. Cech, S. A. Savage, R. A. Reijo Pera and S. E. Artandi (2011). "Telomere shortening and loss of self-renewal in dyskeratosis congenita induced pluripotent stem cells." Nature **474**(7351): 399-402.

Boria, I., E. Garelli, H. T. Gazda, A. Aspesi, P. Quarello, E. Pavesi, D. Ferrante, J. J. Meerpohl, M. Kartal, L. Da Costa, A. Proust, T. Leblanc, M. Simansour, N. Dahl, A. S. Frojmark, D. Pospisilova, R. Cmejla, A. H. Beggs, M. R. Sheen, M. Landowski, C. M. Buros, C. M. Clinton, L. J. Dobson, A. Vlachos, E. Atsidaftos, J. M. Lipton, S. R. Ellis, U. Ramenghi and I. Dianzani (2010). "The ribosomal basis of Diamond-Blackfan Anemia: mutation and database update." Hum Mutat **31**(12): 1269-1279.

Bosch, D. G., F. N. Boonstra, N. de Leeuw, R. Pfundt, W. M. Nillesen, J. de Ligt, C. Gilissen, S. Jhangiani, J. R. Lupski, F. P. Cremers and B. B. de Vries (2016). "Novel genetic causes for cerebral visual impairment." Eur J Hum Genet **24**(5): 660-665.

Brion, M., A. Blanco-Verea, B. Sobrino, M. Santori, R. Gil, E. Ramos-Luis, M. Martinez, J. Amigo and A. Carracedo (2014). "Next generation sequencing challenges in the analysis of cardiac sudden death due to arrhythmogenic disorders." Electrophoresis **35**(21-22): 3111-3116.

Brockschmidt, A., B. Chung, S. Weber, D. C. Fischer, M. Kolatsi-Joannou, L. Christ, A. Heimbach, D. Shtiza, G. Klaus, G. D. Simonetti, M. Konrad, P. Winyard, D. Haffner, F. Schaefer and R. G. Weber (2012). "CHD1L: a new candidate gene for congenital anomalies of the kidneys and urinary tract (CAKUT)." Nephrol Dial Transplant **27**(6): 2355-2364.

Caglayan, A. O., S. Comu, J. F. Baranoski, Y. Parman, H. Kaymakcalan, G. T. Akgumus, C. Caglar, D. Dolen, E. Z. Erson-Omay, A. S. Harmanci, K. Mishra-Gorur, H. H. Freeze, K. Yasuno, K. Bilguvar and M. Gunel (2015). "NGLY1 mutation causes neuromotor impairment, intellectual disability, and neuropathy." Eur J Med Genet **58**(1): 39-43.

Chen, L., M. L. Marquardt, D. J. Tester, K. J. Sampson, M. J. Ackerman and R. S. Kass (2007). "Mutation of an A-kinase-anchoring protein causes long-QT syndrome." Proc Natl Acad Sci U S A **104**(52): 20990-20995.

Choesmel, V., S. Fribourg, A. H. Aguissa-Toure, N. Pinaud, P. Legrand, H. T. Gazda and P. E. Gleizes (2008). "Mutation of ribosomal protein RPS24 in Diamond-Blackfan anemia results in a ribosome biogenesis disorder." Hum Mol Genet **17**(9): 1253-1263.

Cordeddu, V., J. C. Yin, C. Gunnarsson, C. Virtanen, S. Drunat, F. Lepri, A. De Luca, C. Rossi, A. Ciolfi, T. J. Pugh, A. Bruselles, J. R. Priest, L. A. Pennacchio, Z. Lu, A. Danesh, R. Quevedo, A. Hamid, S. Martinelli, F. Pantaleoni, M. Gnazzo, P. Daniele, C. Lissewski, G. Bocchinfuso, L. Stella, S. Odent, N. Philip, L. Faivre, M. Vlckova, E. Seemanova, C. Digilio, M. Zenker, G. Zampino, A. Verloes, B. Dallapiccola, A. E. Roberts, H. Cave, B. D. Gelb, B. G. Neel and M. Tartaglia (2015). "Activating Mutations Affecting the Dbl Homology Domain of SOS2 Cause Noonan Syndrome." Hum Mutat **36**(11): 1080-1087.

de Saint Basile, G., F. Geissmann, E. Flori, B. Uring-Lambert, C. Soudais, M. Cavazzana-Calvo, A. Durandy, N. Jabado, A. Fischer and F. Le Deist (2004). "Severe combined immunodeficiency caused by deficiency in either the delta or the epsilon subunit of CD3." J Clin Invest **114**(10): 1512-1517.

DeJarnette, J. B., C. L. Sommers, K. Huang, K. J. Woodside, R. Emmons, K. Katz, E. W. Shores and P. E. Love (1998). "Specific requirement for CD3epsilon in T cell development." Proc Natl Acad Sci U S A **95**(25): 14909-14914.

Doherty, L., M. R. Sheen, A. Vlachos, V. Choesmel, M. F. O'Donohue, C. Clinton, H. E. Schneider, C. A. Sieff, P. E. Newburger, S. E. Ball, E. Niewiadomska, M. Matysiak, B. Glader, R. J. Arceci, J. E. Farrar, E. Atsidaftos, J. M. Lipton, P. E. Gleizes and H. T. Gazda (2010). "Ribosomal protein genes RPS10 and RPS26 are commonly mutated in Diamond-Blackfan anemia." Am J Hum Genet **86**(2): 222-228.

Enns, G. M., V. Shashi, M. Bainbridge, M. J. Gambello, F. R. Zahir, T. Bast, R. Crimian, K. Schoch, J. Platt, R. Cox, J. A. Bernstein, M. Scavina, R. S. Walter, A. Bibb, M. Jones, M. Hegde, B. H. Graham, A. C. Need, A. Oviedo, C. P. Schaaf, S. Boyle, A. J. Butte, R. Chen, R. Chen, M. J. Clark, R. Haraksingh, T. M. Cowan, P. He, S. Langlois, H. Y. Zoghbi, M. Snyder, R. A. Gibbs, H. H. Freeze and D. B. Goldstein (2014). "Mutations in NGLY1 cause an inherited disorder of the endoplasmic reticulum-associated degradation pathway." Genet Med **16**(10): 751-758.

Fuehrer, M., U. Pannicke, C. Schuetz, E. M. Jacobsen, A. Schulz, W. Friedrich, K. Schwarz and M. Honig (2014). "Successful haploidentical hematopoietic stem cell transplantation in a patient with SCID due to CD3epsilon deficiency: need for IgG-substitution 6 years later." Klin Padiatr **226**(3): 149-153.

Gazda, H. T., A. Grabowska, L. B. Merida-Long, E. Latawiec, H. E. Schneider, J. M. Lipton, A. Vlachos, E. Atsidaftos, S. E. Ball, K. A. Orfali, E. Niewiadomska, L. Da Costa, G. Tchernia, C. Niemeyer, J. J. Meerpohl, J. Stahl, G. Schratt, B. Glader, K. Backer, C. Wong, D. G. Nathan, A. H. Beggs and C. A. Sieff (2006). "Ribosomal protein S24 gene is mutated in Diamond-Blackfan anemia." Am J Hum Genet **79**(6): 1110-1118.

Ghemlas, I., H. Li, B. Zlateska, R. Klaassen, C. V. Fernandez, R. A. Yanofsky, J. Wu, Y. Pastore, M. Silva, J. H. Lipton, J. Brossard, B. Michon, S. Abish, M. Steele, R. Sinha, M. Belletrutti, V. R. Breakey, L. Jardine, L. Goodyear, L. Sung, S. Dhanraj, E. Reble, A. Wagner, J. Beyene, P. Ray, S. Meyn, M. Cada and Y. Dror (2015). "Improving diagnostic precision, care and syndrome definitions using comprehensive next-generation sequencing for the inherited bone marrow failure syndromes." J Med Genet **52**(9): 575-584.

Habibi-Babadi, N., A. Su, C. E. de Carvalho and A. Colavita (2010). "The N-glycanase png-1 acts to limit axon branching during organ formation in Caenorhabditis elegans." J Neurosci **30**(5): 1766-1776.

Hammarsjo, A., A. Nordgren, K. Lagerstedt-Robinson, H. Malmgren, D. Nilsson, S. Wedren, M. Nordenskjold, G. Nishimura and G. Grigelioniene (2016). "Pathogenenic variant in the COL2A1 gene is associated with Spondyloepiphyseal dysplasia type Stanescu." Am J Med Genet A **170a**(1): 266-269.

He, P., J. E. Grotzke, B. G. Ng, M. Gunel, H. Jafar-Nejad, P. Cresswell, G. M. Enns and H. H. Freeze (2015). "A congenital disorder of deglycosylation: Biochemical characterization of N-glycanase 1 deficiency in patient fibroblasts." Glycobiology **25**(8): 836-844.

Heeley, J. and M. Shinawi (2015). "Multi-systemic involvement in NGLY1-related disorder caused by two novel mutations." Am J Med Genet A **167a**(4): 816-820.

Higuchi, Y., J. Shimizu, M. Hatanaka, E. Kitano, H. Kitamura, H. Takada, M. Ishimura, T. Hara, O. Ohara, K. Asagoe and T. Kubo (2013). "The identification of a novel splicing mutation in C1qB in a Japanese family with C1q deficiency: a case report." Pediatr Rheumatol Online J **11**(1): 41.

Huang, C., Y. Harada, A. Hosomi, Y. Masahara-Negishi, J. Seino, H. Fujihira, Y. Funakoshi, T. Suzuki, N. Dohmae and T. Suzuki (2015). "Endo-beta-N-acetylglucosaminidase forms N-GlcNAc protein aggregates during ER-associated degradation in Ngly1-defective cells." Proc Natl Acad Sci U S A **112**(5): 1398-1403.

Hwang, D. Y., G. C. Dworschak, S. Kohl, P. Saisawat, A. Vivante, A. C. Hilger, H. M. Reutter, N. A. Soliman, R. Bogdanovic, E. O. Kehinde, V. Tasic and F. Hildebrandt (2014). "Mutations in 12 known dominant disease-causing genes clarify many congenital anomalies of the kidney and urinary tract." Kidney Int **85**(6): 1429-1433.

Jensen, D. A., A. Steplewski, K. Gawron and A. Fertala (2011). "Persistence of intracellular and extracellular changes after incompletely suppressing expression of the R789C (p.R989C) and R992C (p.R1192C) collagen II mutants." Hum Mutat **32**(7): 794-805.

Jurgens, J., N. Sobreira, P. Modaff, C. A. Reiser, S. H. Seo, M. W. Seong, S. S. Park, O. H. Kim, T. J. Cho and R. M. Pauli (2015). "Novel COL2A1 variant (c.619G>A, p.Gly207Arg) manifesting as a phenotype similar to progressive pseudorheumatoid dysplasia and spondyloepiphyseal dysplasia, Stanescu type." Hum Mutat **36**(10): 1004-1008.
Karaca, E., T. Harel, D. Pehlivan, S. N. Jhangiani, T. Gambin, Z. Coban Akdemir, C. Gonzaga-Jauregui, S. Erdin, Y. Bayram, I. M. Campbell, J. V. Hunter, M. M. Atik, H. Van Esch, B. Yuan, W. Wiszniewski, S. Isikay, G. Yesil, O. O. Yuregir, S. Tug Bozdogan, H. Aslan, H. Aydin, T. Tos, A. Aksoy, D. C. De Vivo, P. Jain, B. B. Geckinli, O. Sezer, D. Gul, B. Durmaz, O. Cogulu, F. Ozkinay, V. Topcu, S. Candan, A. H. Cebi, M. Ikbal, E. Yilmaz Gulec, A. Gezdirici, E. Koparir, F. Ekici, S. Coskun, S. Cicek, K. Karaer, A. Koparir, M. B. Duz, E. Kirat, E. Fenercioglu, H. Ulucan, M. Seven, T. Guran, N. Elcioglu, M. S. Yildirim, D. Aktas, M. Alikasifoglu, M. Ture, T. Yakut, J. D. Overton, A. Yuksel, M. Ozen, D. M. Muzny, D. R. Adams, E. Boerwinkle, W. K. Chung, R. A. Gibbs and J. R. Lupski (2015). "Genes that Affect Brain Structure and Function Identified by Rare Variant Analyses of Mendelian Neurologic Disease." Neuron **88**(3): 499-513.
Kohl, S., D. Zobor, W. C. Chiang, N. Weisschuh, J. Staller, I. Gonzalez Menendez, S. Chang, S. C. Beck, M. Garcia Garrido, V. Sothilingam, M. W. Seeliger, F. Stanzial, F. Benedicenti, F. Inzana, E. Heon, A. Vincent, J. Beis, T. M. Strom, G. Rudolph, S. Roosing, A. I. Hollander, F. P. Cremers, I. Lopez, H. Ren, A. T. Moore, A. R. Webster, M. Michaelides, R. K. Koenekoop, E. Zrenner, R. J. Kaufman, S. H. Tsang, B. Wissinger and J. H. Lin (2015). "Mutations in the unfolded protein response regulator ATF6 cause the cone dysfunction disorder achromatopsia." Nat Genet **47**(7): 757-765.
Kuznetsov, S. G., D. C. Haines, B. K. Martin and S. K. Sharan (2009). "Loss of Rad51c leads to embryonic lethality and modulation of Trp53-dependent tumorigenesis in mice." Cancer Res **69**(3): 863-872.
Li, R. G., Q. Wang, Y. J. Xu, M. Zhang, X. K. Qu, X. Liu, W. Y. Fang and Y. Q. Yang (2013). "Mutations of the SCN4B-encoded sodium channel beta4 subunit in familial atrial fibrillation." Int J Mol Med **32**(1): 144-150.
Li, S. W., D. J. Prockop, H. Helminen, R. Fassler, T. Lapvetelainen, K. Kiraly, A. Peltarri, J. Arokoski, H. Lui, M. Arita and et al. (1995). "Transgenic mice with targeted inactivation of the Col2 alpha 1 gene for collagen II develop a skeleton with membranous and periosteal bone but no endochondral bone." Genes Dev **9**(22): 2821-2830.
Lieve, K. V., L. Williams, A. Daly, G. Richard, S. Bale, D. Macaya and W. K. Chung (2013). "Results of genetic testing in 855 consecutive unrelated patients referred for long QT syndrome in a clinical laboratory." Genet Test Mol Biomarkers **17**(7): 553-561.
Marquart, H. V., L. Schejbel, A. Sjoholm, U. Martensson, S. Nielsen, A. Koch, A. Svejgaard and P. Garred (2007). "C1q deficiency in an Inuit family: identification of a new class of C1q disease-causing mutations." Clin Immunol **124**(1): 33-40.
McAdam, R. A., D. Goundis and K. B. Reid (1988). "A homozygous point mutation results in a stop codon in the C1q B-chain of a C1q-deficient individual." Immunogenetics **27**(4): 259-264.
Medeiros-Domingo, A., T. Kaku, D. J. Tester, P. Iturralde-Torres, A. Itty, B. Ye, C. Valdivia, K. Ueda, S. Canizales-Quinteros, M. T. Tusie-Luna, J. C. Makielski and M. J. Ackerman (2007). "SCN4B-encoded sodium channel beta4 subunit in congenital long-QT syndrome." Circulation **116**(2): 134-142.
Miura-Shimura, Y., K. Nakamura, M. Ohtsuji, H. Tomita, Y. Jiang, M. Abe, D. Zhang, Y. Hamano, H. Tsuda, H. Hashimoto, H. Nishimura, S. Taki, T. Shirai and S. Hirose (2002). "C1q regulatory region polymorphism down-regulating murine c1q protein levels with linkage to lupus nephritis." J Immunol **169**(3): 1334-1339.

Mullally, J., I. Goldenberg, A. J. Moss, C. M. Lopes, M. J. Ackerman, W. Zareba, S. McNitt, J. L. Robinson, J. Benhorin, E. S. Kaufman, J. A. Towbin and A. Barsheshet (2013). "Risk of life-threatening cardiac events among patients with long QT syndrome and multiple mutations." Heart Rhythm **10**(3): 378-382.
Nakanishi, T., M. Shimazawa, S. Sugitani, T. Kudo, S. Imai, Y. Inokuchi, K. Tsuruma and H. Hara (2013). "Role of endoplasmic reticulum stress in light-induced photoreceptor degeneration in mice." J Neurochem **125**(1): 111-124.
Need, A. C., V. Shashi, Y. Hitomi, K. Schoch, K. V. Shianna, M. T. McDonald, M. H. Meisler and D. B. Goldstein (2012). "Clinical application of exome sequencing in undiagnosed genetic conditions." J Med Genet **49**(6): 353-361.
Nicolaou, N., S. L. Pulit, I. J. Nijman, G. R. Monroe, W. F. Feitz, M. F. Schreuder, A. M. van Eerde, T. P. de Jong, J. C. Giltay, B. van der Zwaag, M. R. Havenith, S. Zwakenberg, L. F. van der Zanden, G. Poelmans, E. A. Cornelissen, M. R. Lilien, B. Franke, N. Roeleveld, I. A. van Rooij, E. Cuppen, E. M. Bongers, R. H. Giles, N. V. Knoers and K. Y. Renkema (2016). "Prioritization and burden analysis of rare variants in 208 candidate genes suggest they do not play a major role in CAKUT." Kidney Int **89**(2): 476-486.
Olesen, M. S., T. Jespersen, J. B. Nielsen, B. Liang, D. V. Moller, P. Hedley, M. Christiansen, A. Varro, S. P. Olesen, S. Haunso, N. Schmitt and J. H. Svendsen (2011). "Mutations in sodium channel beta-subunit SCN3B are associated with early-onset lone atrial fibrillation." Cardiovasc Res **89**(4): 786-793.
Petry, F., G. Hauptmann, J. Goetz, E. Grosshans and M. Loos (1997). "Molecular basis of a new type of C1q-deficiency associated with a non-functional low molecular weight (LMW) C1q: parallels and differences to other known genetic C1q-defects." Immunopharmacology **38**(1-2): 189-201.
Pogacic, V., F. Dragon and W. Filipowicz (2000). "Human H/ACA small nucleolar RNPs and telomerase share evolutionarily conserved proteins NHP2 and NOP10." Mol Cell Biol **20**(23): 9028-9040.
Quarello, P., E. Garelli, A. Carando, A. Brusco, R. Calabrese, C. Dufour, D. Longoni, A. Misuraca, L. Vinti, A. Aspesi, L. Biondini, F. Loreni, I. Dianzani and U. Ramenghi (2010). "Diamond-Blackfan anemia: genotype-phenotype correlations in Italian patients with RPL5 and RPL11 mutations." Haematologica **95**(2): 206-213.
Renard, V., L. Ardouin, M. Malissen, G. Milon, M. Lebastard, A. Gillet, B. Malissen and E. Vivier (1995). "Normal development and function of natural killer cells in CD3 epsilon delta 5/delta 5 mutant mice." Proc Natl Acad Sci U S A **92**(16): 7545-7549.
Rodrigue, A., Y. Coulombe, K. Jacquet, J. P. Gagne, C. Roques, S. Gobeil, G. Poirier and J. Y. Masson (2013). "The RAD51 paralogs ensure cellular protection against mitotic defects and aneuploidy." J Cell Sci **126**(Pt 1): 348-359.
Roumenina, L. T., D. Sene, M. Radanova, J. Blouin, L. Halbwachs-Mecarelli, M. A. Dragon-Durey, W. H. Fridman and V. Fremeaux-Bacchi (2011). "Functional complement C1q abnormality leads to impaired immune complexes and apoptotic cell clearance." J Immunol **187**(8): 4369-4373.
Rukavina, I., G. Mortier, L. Van Laer, M. Frkovic, T. Dapic and M. Jelusic (2014). "Mutation in the type II collagen gene (COL2AI) as a cause of primary osteoarthritis associated with mild spondyloepiphyseal involvement." Semin Arthritis Rheum **44**(1): 101-104.
Shigemizu, D., T. Aiba, H. Nakagawa, K. Ozaki, F. Miya, W. Satake, T. Toda, Y. Miyamoto, A. Fujimoto, Y. Suzuki, M. Kubo, T. Tsunoda, W. Shimizu and T. Tanaka (2015). "Exome Analyses of Long QT Syndrome Reveal Candidate Pathogenic Mutations in Calmodulin-Interacting Genes." PLoS One **10**(7): e0130329.

Smeenk, G., A. J. de Groot, R. J. Romeijn, P. P. van Buul, M. Z. Zdzienicka, L. H. Mullenders, A. Pastink and B. C. Godthelp (2010). "Rad51C is essential for embryonic development and haploinsufficiency causes increased DNA damage sensitivity and genomic instability." Mutat Res **689**(1-2): 50-58.
Smetanina, N. S., I. V. Mersiyanova, M. A. Kurnikova, G. S. Ovsyannikova, L. A. Hachatryan, V. O. Bobrynina, M. A. Maschan, G. A. Novichkova, J. M. Lipton and A. A. Maschan (2015). "Clinical and genomic heterogeneity of Diamond Blackfan anemia in the Russian Federation." Pediatr Blood Cancer **62**(9): 1597-1600.
Somyajit, K., S. Subramanya and G. Nagaraju (2012). "Distinct roles of FANCO/RAD51C protein in DNA damage signaling and repair: implications for Fanconi anemia and breast cancer susceptibility." J Biol Chem **287**(5): 3366-3380.
Soudais, C., J. P. de Villartay, F. Le Deist, A. Fischer and B. Lisowska-Grospierre (1993). "Independent mutations of the human CD3-epsilon gene resulting in a T cell receptor/CD3 complex immunodeficiency." Nat Genet **3**(1): 77-81.
Trahan, C., C. Martel and F. Dragon (2010). "Effects of dyskeratosis congenita mutations in dyskerin, NHP2 and NOP10 on assembly of H/ACA pre-RNPs." Hum Mol Genet **19**(5): 825-836.
Troedson, C., M. Wong, J. Dalby-Payne, M. Wilson, M. Dexter, G. I. Rice, Y. J. Crow and R. C. Dale (2013). "Systemic lupus erythematosus due to C1q deficiency with progressive encephalopathy, intracranial calcification and acquired moyamoya cerebral vasculopathy." Lupus **22**(6): 639-643.
van Schaarenburg, R. A., N. A. Daha, J. J. Schonkeren, E. W. Nivine Levarht, D. J. van Gijlswijk-Janssen, F. A. Kurreeman, A. Roos, C. van Kooten, C. A. Koelman, M. R. Ernst-Kruis, R. E. Toes, T. W. Huizinga, A. C. Lankester and L. A. Trouw (2015). "Identification of a novel non-coding mutation in C1qB in a Dutch child with C1q deficiency associated with recurrent infections." Immunobiology **220**(3): 422-427.
Vaz, F., H. Hanenberg, B. Schuster, K. Barker, C. Wiek, V. Erven, K. Neveling, D. Endt, I. Kesterton, F. Autore, F. Fraternali, M. Freund, L. Hartmann, D. Grimwade, R. G. Roberts, H. Schaal, S. Mohammed, N. Rahman, D. Schindler and C. G. Mathew (2010). "Mutation of the RAD51C gene in a Fanconi anemia-like disorder." Nat Genet **42**(5): 406-409.
Venteicher, A. S., E. B. Abreu, Z. Meng, K. E. McCann, R. M. Terns, T. D. Veenstra, M. P. Terns and S. E. Artandi (2009). "A human telomerase holoenzyme protein required for Cajal body localization and telomere synthesis." Science **323**(5914): 644-648.
Vulliamy, T., R. Beswick, M. Kirwan, A. Marrone, M. Digweed, A. Walne and I. Dokal (2008). "Mutations in the telomerase component NHP2 cause the premature ageing syndrome dyskeratosis congenita." Proc Natl Acad Sci U S A **105**(23): 8073-8078.
Xu, M., V. Gelowani, A. Eblimit, F. Wang, M. P. Young, B. L. Sawyer, L. Zhao, G. Jenkins, D. J. Creel, K. Wang, Z. Ge, H. Wang, Y. Li, M. E. Hartnett and R. Chen (2015). "ATF6 Is Mutated in Early Onset Photoreceptor Degeneration With Macular Involvement." Invest Ophthalmol Vis Sci **56**(6): 3889-3895.
Yamamoto, G. L., M. Aguena, M. Gos, C. Hung, J. Pilch, S. Fahiminiya, A. Abramowicz, I. Cristian, M. Buscarilli, M. S. Naslavsky, A. C. Malaquias, M. Zatz, O. Bodamer, J. Majewski, A. A. Jorge, A. C. Pereira, C. A. Kim, M. R. Passos-Bueno and D. R. Bertola (2015). "Rare variants in SOS2 and LZTR1 are associated with Noonan syndrome." J Med Genet **52**(6): 413-421.
Yazaki, M., M. Kamei, Y. Ito, Y. Konno, R. Wang, T. Toki and E. Ito (2012). "A novel mutation of ribosomal protein S10 gene in a Japanese patient with diamond-Blackfan anemia." J Pediatr Hematol Oncol **34**(4): 293-295.

Zhong, F., S. A. Savage, M. Shkreli, N. Giri, L. Jessop, T. Myers, R. Chen, B. P. Alter and S. E. Artandi (2011). "Disruption of telomerase trafficking by TCAB1 mutation causes dyskeratosis congenita." Genes Dev **25**(1): 11-16.
Zhou, X., G. Zhao, J. J. Truglio, L. Wang, G. Li, W. J. Lennarz and H. Schindelin (2006). "Structural and biochemical studies of the C-terminal domain of mouse peptide-N-glycanase identify it as a mannose-binding module." Proc Natl Acad Sci U S A **103**(46): 17214-17219.
